# A fast and cost-effective approach to develop and map EST-SSR markers: oak as a case study

**DOI:** 10.1186/1471-2164-11-570

**Published:** 2010-10-15

**Authors:** Jérôme Durand, Catherine Bodénès, Emilie Chancerel, Jean-Marc Frigerio, Giovanni Vendramin, Federico Sebastiani, Anna Buonamici, Oliver Gailing, Hans-Peter Koelewijn, Fiorella Villani, Claudia Mattioni, Marcello Cherubini, Pablo G Goicoechea, Ana Herrán, Ziortza Ikaran, Cyril Cabané, Saneyoshi Ueno, Florian Alberto, Pierre-Yves Dumoulin, Erwan Guichoux, Antoine de Daruvar, Antoine Kremer, Christophe Plomion

**Affiliations:** 1 INRA, UMR1202 BIOGECO, F-33610 Cestas, France; 2 Université de Bordeaux, UMR1202 BIOGECO, F-33610 Cestas, France; 3 Plant Genetics Institute, National Research Council, Via Madonna del Piano 10, 50019 Sesto Fiorentino (FI), Italy; 4 Forest Genetics and Forest Tree Breeding Büsgen Institute Faculty of Forest Sciences and Forest Ecology Göttingen University, Büsgenweg 2, Göttingen, 37077, Germany; 5 School 07 Forest Resources and Environmental Science, Michigan Technological University, Houghton 49931, Michigan, USA; 6 ALTERRA - Wageningen UR, PO Box 47, Wageningen, 6700 AA, The Netherlands; 7 CNR Istituto di Biologia Agroambientale e Forestale, Porano (TR), 05010, Italy; 8 NEIKER, Dpto Biotecnologia, Vitoria-Gasteiz, 01080, Spain; 9 CBiB - Université Victor Segalen Bordeaux 2 146, rue Léo Saignat, 33076 Bordeaux, France; 10 Forestry and Forest Products Research Institute, Department of Forest Genetics, Tree Genetics Laboratory, 1 Matsunosato, Tsukuba, Ibaraki, 305-8687, Japan

## Abstract

**Background:**

Expressed Sequence Tags (ESTs) are a source of simple sequence repeats (SSRs) that can be used to develop molecular markers for genetic studies. The availability of ESTs for *Quercus robur *and *Quercus petraea *provided a unique opportunity to develop microsatellite markers to accelerate research aimed at studying adaptation of these long-lived species to their environment. As a first step toward the construction of a SSR-based linkage map of oak for quantitative trait locus (QTL) mapping, we describe the mining and survey of EST-SSRs as well as a fast and cost-effective approach (bin mapping) to assign these markers to an approximate map position. We also compared the level of polymorphism between genomic and EST-derived SSRs and address the transferability of EST-SSRs in *Castanea sativa *(chestnut).

**Results:**

A catalogue of 103,000 Sanger ESTs was assembled into 28,024 unigenes from which 18.6% presented one or more SSR motifs. More than 42% of these SSRs corresponded to trinucleotides. Primer pairs were designed for 748 putative unigenes. Overall 37.7% (283) were found to amplify a single polymorphic locus in a reference full-sib pedigree of *Quercus robur*. The usefulness of these loci for establishing a genetic map was assessed using a bin mapping approach. Bin maps were constructed for the male and female parental tree for which framework linkage maps based on AFLP markers were available. The bin set consisting of 14 highly informative offspring selected based on the number and position of crossover sites. The female and male maps comprised 44 and 37 bins, with an average bin length of 16.5 cM and 20.99 cM, respectively. A total of 256 EST-SSRs were assigned to bins and their map position was further validated by linkage mapping. EST-SSRs were found to be less polymorphic than genomic SSRs, but their transferability rate to chestnut, a phylogenetically related species to oak, was higher.

**Conclusion:**

We have generated a bin map for oak comprising 256 EST-SSRs. This resource constitutes a first step toward the establishment of a gene-based map for this genus that will facilitate the dissection of QTLs affecting complex traits of ecological importance.

## Background

Catalogues of Expressed Sequence Tags (ESTs) are developed from cDNA libraries to obtain expressional sequence information in contrasting environmental conditions or across developmental stages. When available, they also offer an inexpensive source of gene-based DNA markers, in particular SSRs [1]. Such collections of ESTs were produced in several plants providing a unique opportunity for searching SSR motifs and further develop the corresponding microsatellite markers [2]. Alternative and promising strategies to develop SSR markers from genome shotgun sequencing have recently emerged with the development of new generation sequencing technologies [3]. However, because ESTs correspond to coding DNA, the flanking sequences of EST-SSRs are located in well-conserved regions across phylogenetically related species, making them markers of choice for comparative mapping and relevant functional and positional candidate genes to study their co-location with quantitative trait loci (QTLs).

The construction of a high resolution genetic map populated with SSRs requires considerable efforts, including the development of several hundreds of markers (depending on the number of linkage groups) and the genotyping of a large number of plants to ensure that most of the markers are correctly ordered, i.e. with a high LOD support for local ordering. Alternatively, bin-mapping or selective mapping [4] offers a less accurate but faster and cost-effective approach to locate many markers on an already existing framework map. This mapping strategy consists of genotyping a subset of highly informative offspring (the bin set) that are selected based on the number and position of crossover sites. In brief, the optimal bin set of a given size presents the maximum number of breaking points evenly spaced throughout the map, ideally resulting in a number of bins that is close to the number of framework marker intervals. This approach has been used successfully in peach [5], melon [6], strawberry [7] and apple [8,9]. Here, we use this approach for the first time in a forest tree species: oak.

Oaks represent a major component of the northern hemisphere forest. In particular, pedunculate *(Quercus robur L.) *oak is widely spread throughout Europe, from Spain to Russia (Ural mountains). This species is associated with important environmental (carbon sequestration, water cycle, reservoir of biodiversity...) and economic (carpentry, furniture, cabinet making, veneer, cask industry, fuel wood, hunting and fungus gathering) services. It has been used for years to study the genetic architecture of forest tree adaptation through common garden experiments [10,11], where natural populations growing in their native environments have been transplanted in a common environment, and QTL mapping studies [12-16], as well as to decipher the molecular mechanisms underlying adaptive traits such as bud phenology [17], water-use efficiency [18] and response to root hypoxia [15].

Different types of molecular markers were developed in *Q. robur *for linkage mapping to study the genetic architecture of adaptive traits. The different versions of the map included hundreds of random amplified polymorphic DNA (RAPD) markers [19], amplified fragment length polymorphisms (AFLP) [12] markers, and a set of 56 simple sequence repeats obtained from enriched genomic libraries (gSSRs) [20]. Because of their highly polymorphic nature and high degree of transferability across species, SSRs proved to be very useful markers to align different maps of *Q. robur *as well as to initiate a comparative mapping analysis with *Castanea sativa *(chestnut), another important Fagaceae species [20,21]. Despite combining interesting features (typically co-dominant and multiallelic, high polymorphism information content, evenly distributed throughout the genome, and high reproducibility) too few SSRs have been yet made available in oak to advance to more detailed genetic studies. The high cost associated with their development from enriched genomic libraries [22] and the lack of sequences for the genus *Quercus *genus probably contributed to the delay of the construction of a large battery of SSRs.

In this context, the main objectives of this study were: i/ to screen the oak ESTs for SSR motifs (*i.e*. type, frequency, and distribution of SSR motifs), ii/ to develop a set of EST-SSR markers and compile the data in a dedicated database, iii/ to compare their polymorphism information content with gSSR, iv/ to test the transferability of these markers in chesnut and v/ to map as much SSR loci as possible on two parental framework linkage maps of *Q. robur *using a bin-mapping approach. This study constitutes the first step toward the establishment of a consensus linkage map for oak based on SSRs segregating in several mapping populations.

## Results

### SSR mining and EST-SSRs frequency

SSRs were searched among the 28,024 unigene elements obtained from the assembly of 103,000 ESTs into 13,477 contigs and 14,547 singletons, using STACKpack™. The search was performed for di- (with a repeat count n ≥ 5 repeat units), tri- (n ≥ 4), tetra- (n ≥ 3), penta- (n ≥ 3) and hexa- (n ≥ 3) nucleotides, using the mreps software [23]. A total of 3,893 unigene elements contained at least one SSRs, resulting into 5,218 microsatellites, ie. a SSR frequency of 18.6%, taking into account multiple occurrences of SSRs in some unigene elements. As expected, the most frequent type of microsatellites corresponded to trimeric SSRs (2,212 unigene elements, i.e. 42% of the detected SSRs). This was followed by dimeric (1,713, 34%) and hexameric (574, 11%) SSRs. The abundance of tetrameric and pentameric SSRs was lower, representing only 8% and 5% of the microsatellites, respectively. The size of the SSR string varied from 10 bp (5 repeats for di-nucleotide motifs) to 132 bp (66 repeats for an AG SSR) and the average number of repeats were 8.8 for dimeric (see additional file 1- table S1 for the distribution), 5 for trimeric (48.8% with 4 repeats), 3.5 for tetrameric (65.6% with 6 repeats), 3.2 for pentameric (81.2% with 3 repeats), and 3.4 for hexameric (72.5% with 3 repeats) SSRs. Among the dimeric SSRs, AG was found as the most common motif (70%), followed by AT (19%), AC (10.5%) and CG (0.1%). Similarly, for trimeric SSRs, the most common motifs were AAG (28%), ACC (14%) and AAC (12.4%). For the three other classes, the most common SSR types corresponded to AAAN (for tetrameric SSRs), AAAAN (for pentameric SSRs), and AAAAAN (for hexameric SSRs). All these SSRs were made available in additional file 1 - table S1, which compiles information such as number of repeats, size of the motif, annotation *etc*.

### Distribution of EST-SSRs

**Figure 1:**
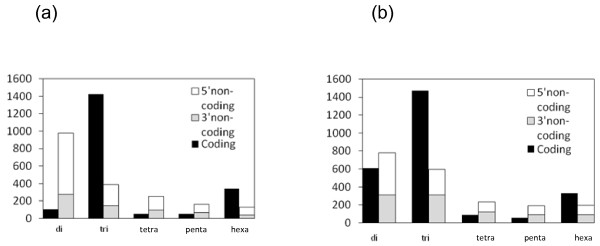
**Microsatellite frequency among coding and 5' and 3' non-coding regions by ESTScan (a) and FrameDP (b)**.

For 86% of the 5,218 SSRs, ESTscan [24] succeeded in estimating whether SSRs were located in non-coding (untranslated) (41.8%, including 21.5% di-, 8.5% tri- 2.8% hexa-SSRs) vs. coding (translated) (43.3%, including 2.2% di-, 31.3% tri- 7.5% hexa-SSRs) regions of each EST. The occurrence of each category in coding and non-coding regions is shown in Figure 1a. Overall, 67.3% and 32.7% of the non-coding SSRs were located at 5'- and 3'-UTR, respectively. Using FrameDP, 83% of the 5,218 SSRs was estimated in at least one predicted peptide (Figure 1b). As ESTScan, FrameDP prediction showed that smaller numbers of SSRs were located in non-coding (37.4%, including 14.6% di-, 11.1% tri- 3.7% hexa-SSRs) compared to coding regions (47.9%, including 11.4% di-, 27.5% tri- and 6.2% hexa-SSRs). Overall, 53.8% and 46.2% of the non-coding SSRs were located at the 5'- and 3'- UTRs, respectively. The most remarkable result obtained by FrameDP was the increased ratio of SSRs predicted in coding regions (from 43.3% to 47.9%), that can be attributed to a higher frequency among dinucleotide motifs compared to ESTscan.

### Marker development

**Figure 2:**
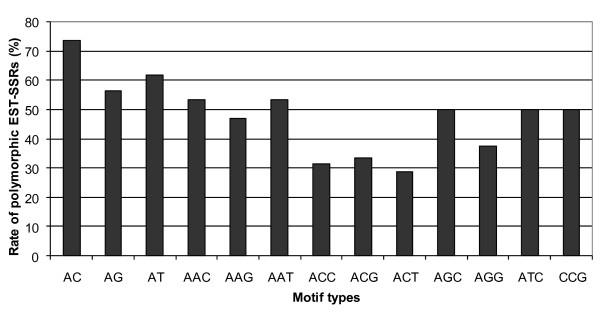
**Rate of polymorphism for different types of di- and tri- SSRs**.

Of the 5,218 SSRs motifs identified, we designed primer pairs for 748 SSRs (additional file 2 - table S1), including 348 di-, 320 tri-, 2 tetra-, 1 penta-, and 77 hexa-nucleotide SSRs. Locus ID, forward and reverse primer sequences, type of motif and length, amplification and polymorphism in the tested full-sib pedigree have been reported in additional file 3 - table S1. A total of 568 primer pairs (75.8%) amplified a PCR product, among which 283 (154 di-, 107 tri-, 1 tetra-, 1 penta- and 20 hexa-nucleotide SSRs) were found to amplify a single polymorphic locus, i.e. 37.7% of the total number of tested primers. It was also found that the level of polymorphism depended on the type of motif (Figure 2). These loci segregated in the testcross configuration, i.e. 1:1 ratio (65 loci in the male and 77 loci in the female parent), or in the intercross configuration, i.e. 1:1:1:1 ratio (135 loci in both parents) or 1:2:1 ratio (6 loci in both parents). Markers segregating 1:1:1:1 were recoded in the 1:1 ratio in the male and female parents.

### Transferability of EST-SSRs

A subset of oak EST-SSRs were also tested for their transferability in chestnut (*Castanea sativa*) another important Fagaceae species. A total of 100 dinucleotide EST-SSRs were tested for their amplification on two DNA specimen (additional file 4 - table S1), from which 63% amplified a single PCR product, a figure that is significantly higher than that obtained for the transferability of dinucleotide genomic SSRs from oak to chestnut, i.e. 47% in [20]. In addition, electronic PCR was carried out against unigene elements for *Quercus mongolica *(Qm) [25] and *Castanopsis sieboldii *(Cs) [26]. There were 52 oak primer pairs that amplified Qm with no mismatch and product size similar to that for European oaks. Six primer pairs amplified two different Qm sequences. For Cs, there were 18 primer pairs that can amplify Cs with no mismatch. One primer pair amplified two different Cs sequences. Seven primer pairs produced ePCR products for both Qm and Cs. Three primer pairs in the present study targeted three unigene elements for which SSR markers were already developed for Qm.

### Comparison between genomic and EST-derived SSRs

A total of 16 dinucleotide genomic SSRs from Alberto *et al*. [27] and 16 dinucleotide EST-SSRs (from this study) were genotyped on the same set of 288 *Q. petraea *genotypes described in [27]. The comparison (taking into account heterogeneous sample size using the rarefaction methods from El Mousadik and Petit, [28] of genetic diversity (He) and allelic richness (A) showed that gSSRs were more polymorphic (He = 0.82 A = 4.34) than EST-SSRs (He = 0.77 and A = 3.78). Other diversity statistics as the size range of the SSR motifs and the number of alleles confirmed the lower level of polymorphism of EST-SSRs compared to gSSRs. The size of the SSR motif was on average 46.75 bp for gSSRs and 26.25 bp for EST-SSRs. The total number of alleles present in the tested population, regardless of their frequency was 21.06 vs. for gSSRs and 12.25 bp for EST-SSRs

### Bin mapping

The two parental maps established by Saintagne *et al*. [12] using Mapmaker 2.0 [29] were first reconstructed (Figure 2) using Joinmap v4.0 [30] based on the same 128 framework markers and 278 progenies. The female map was covered by 38 AFLPs, 6 RAPDs and 28 gSSRs resulting in 63 marker intervals spanning 728.8 cM. The male map was divided by 60 marker intervals and comprised 43 AFLPs, 4 RAPDs and 23 gSSRs for a total map length of 776.9 cM. Each linkage map consisted in 12 linkage groups that corresponded to the number of haploid chromosomes in oak. Compared to the map previously constructed using Mapmaker, very few differences were noticed, consisting mainly in few inversions (ZQR5a and E-AAC/M-CAC-202/3 on LG8F, E-AAG/M-CTA-150/5 and E-AAC/M-CTT-120 on LG4M) and three unlinked markers (E-AAG/M-CTT-168 on LG10F, and E-AAG/M-CTT-363 on LG10M and P-CCA/M-ATA-335 on LG12M). The total map lengths were however quite different (929 vs. 728.8 cM for the female map and 890 vs. 776.9 cM for the male map, using Mapmaker and Joinmap, respectively). Similar results have been reported elsewhere (e.g. [31] and [32]) and is attributed to the method used by the software to calculate Kosambi genetic distances.

Using the bin set of 14 offsprings, the framework maps were divided into 44 and 37 bins resulting in an average bin length of 16.5 cM and 20.9 cM for the female and male map, respectively. Double crossing-overs were taken into account to define the bin set in order to minimize the effect of possible genotyping errors. The longest bins identified spanned 38.1 cM (bin 10.2) for the female and 79.9 cM (bin 5.1) for the male map. On average, there were 1.88 and 1.80 different genotypic points between contiguous bins in the female and male maps. Therefore, more genotypic combinations might exist to fit within intermediate positions.

**Figure 3:**
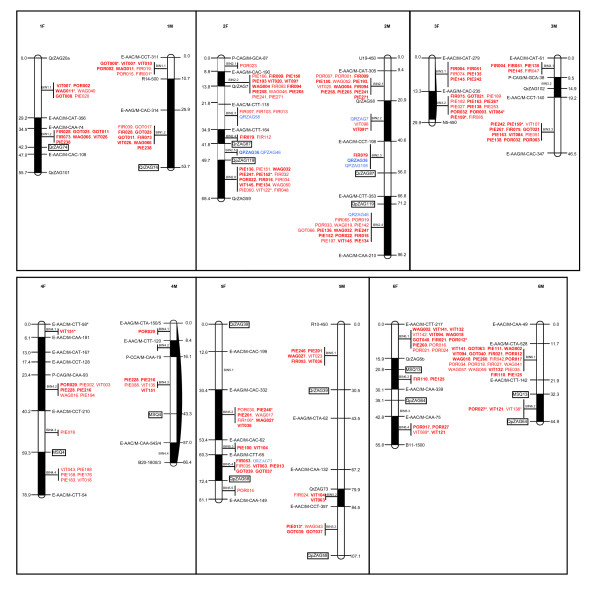
**Bin position of EST-SSRs for linkage groups 1 to 6**. In black: framework markers (AFLP, RAPD, gSSR), in red: EST-SSRs, in blue: gSSRs, squared: fully informative gSSR framework markers. An asterisk indicates SSRs with ambiguous position. Bold type indicates fully informative EST-SSRs and gSSRs. F: female map, M: male map.

**Figure 4:**
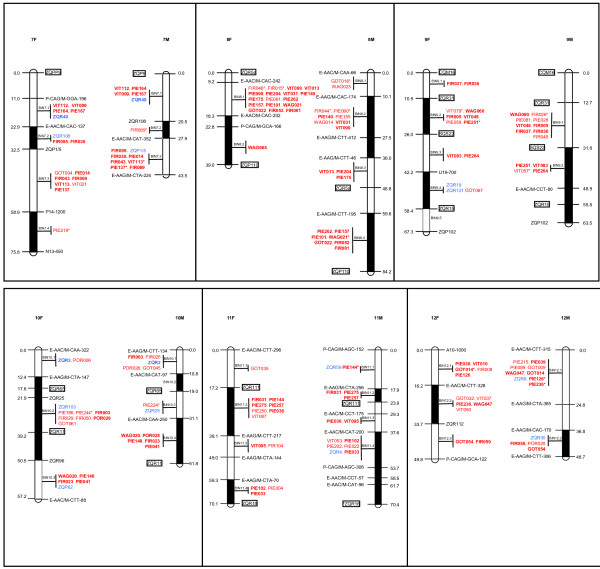
**Bin position of EST-SSRs for linkage groups 7 to 12**. In black: framework markers (AFLP, RAPD, gSSR), in red: EST-SSRs, in blue: gSSRs, squared: fully informative gSSR framework markers. An asterisk indicates SSRs with ambiguous position. Bold type indicates fully informative EST-SSRs and gSSRs. F: female map, M: male map.

A total of 283 polymorphic EST-SSRs were genotyped on the bin set and the parents of the full- sib pedigree (Figures 3, 4). Overall 256 markers were assigned by graphical genotyping (i.e. graphical representation of genotypic information for individual genotypes as defined by Young and Tanksley [33]) to their respective bin. The remaining 27 markers corresponded either to markers segregating 1:2:1 (6 loci) or presented ambiguous bin positions (21 loci) and were therefore left out from the analysis. On the female map, 198 markers were assigned to bins, giving an average of 4.5 markers per bins ranging from 0 (bin 5.1, 6.3, 9.5) to 18 (bin 2.6). On the male map, 185 markers were assigned to bins, giving an average of 5 markers per bin ranging from 0 (bin 3.2, 4.2, 4.4, 10.2) to 22 (bin 6.1). Overall, EST-SSRs were evenly distributed across the linkage groups. More precisely, respectively 69 and 78 markers for the female and the male map presented exactly the same genotypic information as bin framework markers, i.e. these markers were positioned at the same location as the markers used for the definition of bins. The others, 104 and 86 markers in the female and in the male map, respectively, were positioned in the bins, presenting a genotype that was compatible with an intermediate bin between two successive bin markers. This is likely the result of large average bin size defined over low marker density framework maps. Only 25 and 21 markers in the female and male maps were involved in one or more double crossing-overs, respectively. Their genotypes were double checked, confirming this observation. These markers were visually assigned to their most probable bins.

### Validation of bin assignment

To test the efficiency of bin mapping, we first compare the known map location of 19 accessory gSSRs (blue type in Figures 3, 4) from the map constructed by Barreneche et al. [20], to their bin positions inferred from the graphical genotyping of 14 F1s. In all cases, both approaches agreed (additional file 5 - table S1), i.e. markers were located either on the same bin (18 markers of class A according to the categories presented in the methods section) or an adjacent bin (1 marker of class B: ZQR49). An *a posteriori *validation was also performed for 146 EST-SSRs (on the female map 47 markers corresponding exactly to bin markers and 54 markers characterized with ambiguous position, on the male map 47 markers corresponding exactly to bin markers and 47 markers characterized with ambiguous position) genotyped on 46 progenies. On the female map, 77 markers showed identical positions between bin assignment and map location (class A), 12 were located in an adjacent bin (class B), 1 was mapped on the same linkage group (class C), and 11 presented a LOD score for linkage < 2 (class D). Overall, the bin assignment was validated for 89% of the markers (class A+B). For the male map, 72, 11, 0 and 11 markers were of class A, B, C and D, respectively, corresponding to a validation rate of 88%. A slightly higher validation rate was obtained for another set of 65 EST-SSRs (53 inter-cross, 7 female and 5 male test-cross markers) genotyped on 92 offsprings, i.e. 98.3% on the female map (53 A, 6 B and 1 D markers), 94.8% on the male map (51 A, 2 B, 2C and 3 D markers).

### Macro-synteny and colinearity

About the conservation of macro-synteny between the male and female maps, it should be noticed that all the 129 inter-cross markers (indicated in bold in Figures 3, 4) were found on homologous linkage groups. A conserved macro-colinearity was also verified based on the 55 inter-cross markers (21 gSSRs and 34 EST-SSRs) genotyped on the extended set of 92 progenies. These markers presented the same order on both maps as illustrated in additional file 6 - figure S1, but with one exception on LG9. Given the number of comparisons, 2 occurrences with different orders were expected by chance alone at a 5% type I error rate. This investigation also provided the opportunity to test whether the male and female gametes presented different levels of recombination. Based on 33 intervals flanked by the same adjacent markers in the male and female maps, no statistical difference was found using a t-test for paired comparisons (data not shown).

## Discussion

### Frequency, distribution and polymorphism of the oak EST-SSRs

EST-derived SSRs have been searched for many years in plant, animal and microbial species. Despite a lower rate of polymorphisms compared to genomic SSRs (confirmed in the present study), EST-SSRs offer a number of advantages over genomic SSRs [2]: (i) their development requires no investment in *de novo *sequencing; (ii) they detect variation in the expressed portion of the genome; (iii) the conservation of primer sites makes them readily transferable across closely related species as illustrated here between oak and chestnut; and (iv) in most cases they can be exploited for population genetic analysis [1].

The number of SSRs detected in ESTs largely depends on the size of the EST catalogue, the algorithm [34] and criteria (type of repeat motif and minimum number of repeat units) used to detect SSR-containing sequences. It is therefore difficult to conclude about the percentage of genes harbouring SSR motifs. This is apparent from several studies: (i) in *Oryza sativa *40.4% [35] and 50% [36] of EST-SSRs were detected using different software and criteria; (ii) Kumpatla and Mukhopadhyay [37] analysed 1.5 million ESTs derived from 55 dicotyledonous species and found that 2.6 to 16.8% of ESTs contained at least one SSR; and (iii) because the level of polymorphism is positively correlated with the length of the repeats region (see next paragraph), some authors have chosen to use more stringent criteria (i.e. increase the minimum number of repeat units in the detection phase) to increase the probability to find polymorphic SSR markers.

The availability of several genome sequences in angiosperms makes it possible to more accurately estimate the proportion of gene models harbouring SSRs in transcribed and UTR regions. In poplar for example, about 6,000 SSRs were found in coding regions and UTRs [38]. Therefore, taking into account the 45,000 putative protein-coding genes [39], 13.4% of the genes would present a SSR. In *Arabidopsis thaliana*, 44% of the 27,158 putative genes contain one or more SSRs [40], but this figure also includes non transcribed regions.

In oak we found that 18.6% of the unigenes presented at least one SSR motif. In two other Fagaceae species, *Quercus mongolica *[25] and *Castanopsis sieboldii *[26] and it was found that 11.8% and 12.8% of the putative unigenes presented microsatellite motifs (from di- to tetra-nucleotide repeats). Taking into account only di-, tri- and tetra-nucleotide repeats, these figures are very similar to our finding (13.4%), although the detection parameters were different (9 for di-, 6 for tri-, 5 for tetra-nucleotides). Also in terms of the abundance of motif types, our study agrees to that of Ueno *et al*. [25,26] and other studies performed in dicotyledonous species (reviewed by Kumpatla and Mukhopadhyay [37]), i.e. AG and AAG were the most abundant di- and trimeric SSRs, respectively. The extremely low number of SSR motifs containing C and G (2 CGs out of 1,713 dimeric SSRs and 103 CCGs out of 2,212 trimeric SSRs) could be attributed to the composition of dicot genes being less rich in G+C compared to monocots due to codon usage bias [41] and to the intrinsic negative correlation between GC content and slippage rate [42].

As expected, the most frequent SSR class corresponded to trinucleotides (42%). This suggests that many of the detected EST-SSRs are in protein-coding regions because changes in trinucleotide repeat number will not cause frame shifts unlike changes in other types of motifs [43]. Indeed, the analysis of the distribution of the EST-SSRs clearly showed that this type of SSR was frequently found (ranging from 27.5% to 31.3% based on FrameDP or ESTscan analysis, respectively) in coding regions in contrast to other SSRs. As for dimeric SSRs, the second most abundant type, our results confirm what has been obtained in other studies, i.e. they were mostly located in non-coding regions, despite a noticeable difference obtained between FrameDP (14.6%) and ESTscan (21.5%). Overall, it should also be noticed that most of the EST-SSRs found in non-coding region were located in the 5' UTR (ranging from 53.8% to 67.3% based on FrameDP or ESTscan analysis, respectively). Higher density of SSR in the 5' UTR was also found in rice [44]. This result could be attributed to either a technical bias (ESTs being mainly generated from their 5'-ends) or a biological feature of plant genes as discussed by Grover et al. [44] and Fujimori et al. [45]. These authors found that rice and Arabidopsis genes presented a higher rate of SSRs in the 5' flanking regions of the genes and interpreted this finding as a regulatory role in gene expression.

To further explore the accuracy of FrameDP and ESTscan results, we carried out a complementary analysis using poplar full length cDNAs for which structural annotations were available [46]. The result of this analysis is provided as supplemental data (additional file 7 - figure S1). By comparing the SSR location based on true structural annotations it was clearly shown that ESTscan performed better than FrameDP, the later over-estimating the presence of dinucleotide motifs in coding regions as was found with the oak data. In agreement with the data reported in rice and Arabidopsis, it was also found that SSRs were more frequent in the 5'UTR of poplar genes (additional file 7 - figure S1).

A total of 748 primer pairs were designed and tested on a set of 4 genotypes, among which 568 (75.8%) yielded amplicons. The failure for 24.2% of the primers to generate an amplicon can be explained: i/ by the presence of large intronic regions preventing genomic DNA to be amplified, ii/ the presence of SNPs/INDEL variation in the priming site of the tested genotypes, preventing the hybridization between the primers and the target DNA, iii/ by the fact that a single PCR program was used without further optimisation, iv/ because the M13 tail (that was added to each forward primer) may interfer with appropriate PCR amplification [47], and v/ because primers could have been designed for chimeric unigene elements. A large proportion (285 out of 568, i.e. 50%) of the successful primer pairs were either monomorphic (163 EST-SSRs) or produced multibanding patterns or yielded faint amplification (122 EST-SSRs), thereby preventing the development of single copy SSRs. This study reveals that polymorphic SSRs (283 loci) tended to have a higher number of repeats (based on the EST data), ie. 10.58 for di, 7.27 for tri- and 3.4 for hexa-SSRs, compared to monomorphic ones (163 loci), i.e. 9.80 for di-, 6.29 for tri-, and 3.20 for hexa-SSRs. The effect of repeat number and motif on the polymorphism was surveyed using logistic regression model by the R software v. 2.6.2 (R Development Core Team 2008), and the effect of repeat number was highly significant (estimate of correlation coefficient for repeat number = 0.237 and P < 0.001). This result agrees with the significant positive correlation that was found between SSR length and polymorphism rate in plants and animals [48].

In oak, polymorphic markers were not evenly distributed among repeat classes, amounted to 58.7%, 44.3% and 36% for di- tri- and hexa- repeats, respectively. These figures confirm the higher level of polymorphism of dinucleotide repeats among plants [49-51]. The lower level of polymorphism for tri- and hexa- SSRs is mainly related to their location in translated sequences compared to dimeric SSRs that were preferentially distributed in UTRs. These observations suggest that natural selection limit both the number and polymorphism rate of SSRs in translated regions of the genes. Moreover, a closer examination among perfect di-and tri- oak SSRs showed that the level of polymorphism (Figure 2) depended on the type of motif. In particular, SSR markers with dinucleotide AC were the most polymorphic loci. These considerations should be taken into account for the development of additional polymorphic SSRs in oak that are conserved among the Fagaceae species, comparative genomics being our ultimate goal. In that respect, we showed that oak dinucleotide EST-SSRs were highly transferable to European chestnut.

### Bin mapping

Linkage mapping is a time consuming process that requires large size recombinant populations (from which progenies are randomly chosen) to locate polymorphic markers onto a genetic map. Other methods that do not rely on meiotic recombination have also been developed to assign any genes to chromosomal locations, such as the use of aneuploid and deletion stocks in polyploids or radiation hybrid panels. One important advantage of these methods is that any sequence of interest is readily placed on a radiation hybrid or deletion map. In contrast, only polymorphic markers can be mapped on a genetic map. However, such approaches have been limited to a handful of plant species, including wheat [52,53]. Alternatively, a computational method was developed [4] to optimize the construction of high-density linkage maps using a reduced sample of selected offsprings presenting complementary recombinational events throughout the genome. A prerequisite to such selective/bin mapping approach is the availability of a high-confidence framework map. The first bin mapping approach was recently implemented in peach [5]. Using only 6 F_2 _progenies, their F_1 _hybrid parent and one of the grand-parental lines, these authors successfully assigned 264 SSRs to 67 bins of the peach map. The bin mapping strategy was also used in melon (121 SSRs/14 plants [6]; 200 SNP-based markers/14 plants [54]), apple (31 SSRs/14 plants [8]) and strawberry (103 SSRs/8 plants [7]).

A bin mapping approach was developed for the first time in a forest tree species to increase the density of SSR markers in the oak linkage map and provide orthologous anchor markers for comparative mapping within the Fagaceae. The selection of the bin set combined the use of Mappop software and visual inspection of the data. It resulted in the selection of 14 plants, which was considered as a suitable size, as a set of 16 samples (14 F1s and both parents) fits in standard 96-well PCR plates. With this subset, 44 (for the female map) and 37 (for the male map) bins were obtained. As expected based on the number of different genotypic points between adjacent bins, about half of the markers presented a genotype that was compatible with a putative bin between two contiguous bins. To investigate the accuracy of the bin mapping approach, a large number of EST-SSRs was genotyped on an extended set of genotypes (46 or 92 F1s). Most markers assigned to bins or putative bins were placed in the expected position, validating the bin mapping strategy for oak, despite the low number of bins compared to similar studies [5,6]. At this stage, it is difficult to propose a general guideline for further bin mapping studies, but some general recommendations can be made: i/ Number of individuals to be included in the bin set: it largely depends on the population and marker types. For instance, there are more genotypic informations in F2s as compared to F1s for codominant markers (3 *vs*. 2 genotypic classes, respectively). Therefore, less individuals will be needed to define the bins with F2 genotypes. It also depends on technical constraints, 14 individuals emerging as a magic number in the few bin mapping studies published so far in plants, since 16 samples, corresponding to 14 offsprings and two parental lines, fits well in a single raw of a 384-well microtiter plate!, ii/ Number of bins: it obviously depends on the number of linkage groups and on the number of individuals included in the bin set (i.e. the more individuals, the more number of bins).

## Conclusion

In the present study we used an EST catalog produced for *Quercus petraea *and *Q. robur*, to mine and develop EST-derived SSRs. We observed a relatively high abundance of single sequence repeats in the oak transcriptome, 18.6% of the unigene elements harboring at least one SSR. Despite being less polymorphic than gSSRs, their many advantages make them markers of choice for genetic analyses. In particular, these functional markers directly sample variations in genes, which enhance their value for analyzing the genetic basis of forest tree adaptation through the use of non neutral, so called "functional" markers in genetic diversity analysis, QTL and association mapping studies as well as comparative genomics.

The present study contributed 283 gene-derived microsatellite markers, 255 of which were efficiently assigned to a bin position using 14 informative individuals. The development and distribution of this reference set of highly recombinant genotypes to the "European oak mapping community" has been instrumental for the development and mapping of this new set of high quality markers that also proved to be useful in a related species (chestnut).

## Methods

### Plant material and DNA extraction

The bin set and the verification panel were selected from the *Quercus robur *full-sib family (3PxA4) described by Saintagne *et al*. [12] The population that was used to compare the level of polymorphism between genomic SSRs and EST-SSRs is described by Alberto *et al*. [27]. DNA was extracted from leaves using DNeasy plant mini kit (Qiagen, Hilden, Germany).

### EST-SSRs detection

SSR motifs (5, 4, 3, 3, and 3 repeats at least for di-, tri-, tetra-, penta- and hexa-nucleotides, respectively) were searched within the first version of the oak unigene set established from the assembly of 103,000 ESTs (available at EMBL). These ESTs were derived from about 20 cDNA libraries constructed from mRNA extracted from 4 tissues (bud, leaf, xylem and root) collected on *Q. robur *and *Q. petreae *genotypes. The main objective to generate such a large number of ESTs was to catalogue as many as possible non-redundant genes (unigene set) of oak. These ESTs were assembled to avoid redundancy in SSR detection using the transcript reconstruction system stackPACK™ [55] from the SAMBI Institute. This pipeline uses the following programs: Cross_Match [56] to clean up the sequences, d2_cluster [57] to perform a loose first stage clustering, PHRAP [58] to assemble these clusters into contigs and finally CRAW [59] to generate the longest consensi.

SSRs motifs were searched using mreps (v. 2.5) [23]. In a comparative study in *Pinus pinaster *(G. Le Provost, unpublished) mreps was found to be more stringent compared to SSRIT [60] and Sputnik v1.22 (http://abajian.net/sputnik/). Once detected, SSRs located 35 nucleotides from either end of each unigene element were discarded to keep enough sequence information for primer design. In addition, those SSRs that were immediately adjacent to each other (separated by less than 30 nucleotides) were merged into a single SSR. The output of mreps was converted into a standard csv file corresponding to the SSR database structure put in place in the frame of the Evoltree project. Specific information for each SSR included the unigene element ID and the annotation, the repeat motif, its length and position (additional file 3 - table S1, also available through the *Quercus *portal (https://w3.pierroton.inra.fr:8443/QuercusPortal/Home.jsf).

ESTscan [24] and FrameDP [61] were used to estimate the location of a coding region within unigenes. By combining the output from mreps, the location of EST-SSR (either coding or noncoding regions) was estimated. Microsatellites, for which no results were returned by each software or location was covered across both coding and non-coding regions, were discarded. Because there are no annotated full-length genes available for oak yet, we used *Arabidopsis thaliana *sequences as a training set for the analysis performed by ESTScan. The resulting matrix was used for peptide prediction of oak unigenes. For the analysis using FrameDP, no specific training set is required.

### SSR genotyping

Primer pairs were designed for 748 unigene elements (including 348 di-, 320 tri-, 2 tetra-, 1 penta-, 77 hexa-nucleotides) using Primer3 [62]. A M13 tail (TGT AAA ACG ACG GCC AGT) [63] was added to the 5'-end of the forward primer to facilitate exchange of primers between the partners of the network that used different capillary electrophoresis systems: i.e. ABI3730 (Applied Biosystems, Carlsbad, CA, USA), Licor 4300 (Licor, Lincoln, NB, USA), Megabace (GE Healthcare, Buckinghamshire, UK). PCR reactions were performed in a final volume of 10 μL containing: 1× PCR-buffer [10 mM Tris-HCl, 50 mM KCl 1.5 mM MgCl_2_, pH 8.3 at 25°C] (BioLabs, Ipswich, England), 100 μM of dNTPs, 0.045 μM of forward primers, 0.165 μM of reverse primer (5 μM), 0.165 μM of M13 primer, 0.25 U of Taq polymerase (BioLabs) and 6 ng of plant DNA. The cycling conditions were as described by Shuelke *et al *[60]: i.e., a first denaturation at 94°C during 4 minutes, 35 cycles at three temperatures, 94°C for 30 s, 56°C for 45 s, and 72°C for 45 s. Additionally 9 cycles were run at 94°C for 30 s, 53°C for 45 s, and 72°C for 45 s and a final extension at 72°C for 10 minutes and a cooling at 10°C. Data generated were analysed using the GeneScan 3.7 and Genotyper 3.7 softwares for ABI, 4300 DNA analyser software for Licor and Fragment Analyser version 1.2 for MegaBace sequencing machine.

### Nomenclature of the markers

EST-SSR marker ID consisted of: three letters to identify the lab where they were developed i.e, PIE for those designed in Pierroton (INRA, France) followed by a serial number. Genomic markers were designated according to the restriction enzymes and the primer combination used, and their amplification size. RAPD markers were named as follows: the letter and the first digit refers to the identification of the OPERON primers [64] and the last digits correspond to the molecular weight of the polymorphic bands.

### Bin mapping strategy

A total of 748 primer-pairs were tested for amplification and polymorphism on both parental trees and two progenies. Given the relatively high number of putative markers, a bin mapping approach was followed (summarized in additional file 8 - figure S1) with the main objective of minimizing the number of trees to be genotyped, while assigning the markers to their most probable map location. From the initial dataset (278 F1s × 953 markers) a double screen was first applied, consisting of selecting individuals with < 50% missing data and markers with a LOD support for local ordering ≥3 (i.e. framework markers according to Saintagne *et al*. [12]), resulting in a total of 66 individuals and 128 testcross (1:1 segregation) and intercross (1:1:1:1 segregation recoded as 1:1 in each parent) markers. Male and female framework maps were then generated under the two-way pseudo-testcross mapping strategy [65] using the regression mapping algorithm of Joinmap v4.0 [30]. These two datasets were used to select a smaller number of highly recombinant progenies as follows: i/ a first set of 46 plants was selected based on maximizing the number of breakpoints along the 24 linkage groups (12 in the male and 12 in the female maps), using the Mappop software [4,66], and ii/ a final subset of 14 F1s (the bin set: #109, #110, #116, #121, #127, #128, #131, #151, #162, #165, #166, #172, #176, #196) was retained by visual inspection, combining three additional criteria: i) selection of individuals with missing data < 10% and presenting a minimum of duplicated bins; ii) optimisation of both female and male map coverage with the smallest bin size as possible, and iii) minimization of double crossing-over between adjacent framework markers. The bin set (and the parental lines) were finally genotyped for all "mappable" markers segregating in testcross (1:1 ratio), intercross (1:2:1) and outcross (1:1:1:1 ratio) configurations. The EST-SSRs were assigned to their most probable bin by matching their genotypic profile to that of the framework markers. Bins were coded by a two-digit number, the first corresponding to the linkage group ID (1 to 12) and the second to their numerical order.

### Validation of bin assignment

To further test the efficiency of the bin mapping approach, we compared the bin location (obtained as described above) with the map location of SSRs. The map position was estimated on an extended set of genotypes using the two-point test for linkage implemented in Joinmap. An *a priori *validation was first carried out based on 19 genomic SSRs (indicated in blue in Figure 2) that were already genotyped and mapped by Barreneche *et al*. [20]. An *a posteriori *validation was also performed for 146 and 65 non-overlapping EST-SSRs that were genotyped on 46 and 92 progenies, respectively. Markers presenting a LOD score for linkage > 2 (for 46 F1s) or 3 (for 92 F1s) were classified into three categories: class A for markers for which the nearest framework marker (FM) was included in the bin, class B for markers for which the nearest FM was found in an adjacent bin, and class C for markers for which the nearest FM was located in a more distant bin or else in another linkage group. Markers presenting a LOD score for linkage below these thresholds were classified as D marker.

### Genetic diversity analysis

Genetic diversity statistics (gene diversity He [67]) and allelic richness (A) were estimated for 16 genomic and 16 EST-derived SSRs using the program Fstat 2.9.3.2 [68]. Allelic richness (A) was calculated using the rarefaction method developed by El Mousadik and Petit [28].

## Authors' contributions

This article is a part of JD's PhD thesis supervised by CB and CP. The idea of the study was developed by CB, AK and CP. AK coordinated the Evoltree project than funded this research. CB coordinated the present study. Marker development was carried out within the Evoltree network by JD, EC, GV, AB, FS, CM, MC, OG, HPK, FV, CM, MC, PGG, AH, ZI. The writing of the manuscript was performed by JD and CP. JD conducted the bin mapping approach and the verification steps. SU performed the ESTscan and Frame DP analysis. The bioinformatics was performed by JMF (EST assembly) and CC (SSR search and databasing) in covariation with JD. AdD supervised the work of CC. FA, PYD, EG, CB and AK performed the diversity analysis. GGV, FS and AB performed the transferability analysis. All the authors read and approved the final version of the manuscript.

Accession numbers for *Quercus robur *and *Quercus petraea *ESTs can be obtained by searching the EMBL database with keyword for organism name "quercus".

## Supplementary Material

Additional file 1 **Table S1**. Occurrence of non-redundant SSRs in the oak unigene, according to the SSR motif and number of repeats. Click here for file

Additional file 2 **Table S1**. Characteristics of the *Quercus *EST-SSRs. Click here for file

Additional file 3 **Table S1**. SSR database. Click here for file

Additional file 4 **Table S1**. Transferability of dinucleotide EST-SSRs from oak to chesnut. Click here for file

Additional file 5 **Table S1**. Segregation, bin and map position of *Quercus *gSSRs and EST-SSRs. Click here for file

Additional file 6 **Figure S1**. A macrosynteny map for oak based on 55 intercross SSRs. In black: framework markers (AFLP, RAPD), in red: EST-SSRs, in blue: gSSRs. Bold types indicate fully informative SSRs. Female linkage groups on the left (F), male linkage group on the right (M). Click here for file

Additional file 7 **Figure S1 Location of EST-SSRs based on FrameDP (a), ESTscan (b) and structural annotation (c) for a set of 4,664 poplar genes**. Methods. 1. 4,664 full-length cDNA sequences of poplar, downloaded from Genbank. 2. SSRs searched using mreps program with default parameters. 3. Coding sequences estimated by FrameDP and ESTScan. A matrix based on Arabidopsis CDS was used for ESTScan. 4. SSR location (coding or non-coding) inferred by combining FrameDP and mreps results (Figure S1a) and ESTScan and mreps results (Figure S1b). SSR locations were also determined using mreps results and structural annotation for the corresponding cDNA (Figure S1c). Results. Figure S1a: SSR location based on the estimation by FrameDP. Figure S1b: SSR location based on the estimation by ESTScan. Figure S1c: SSR location based on structural annotation. Click here for file

Additional file 8 **Figure S1**. Schematic representation of the bin mapping strategy. Click here for file
