# Supplementary figures and images for: A fast and cost-effective approach to develop and map EST-SSR markers: oak as a case study
Source: BMC Genomics. 2010 Oct 15;11:570. doi: 10.1186/1471-2164-11-570 (PMC3091719; doi:10.1186/1471-2164-11-570)

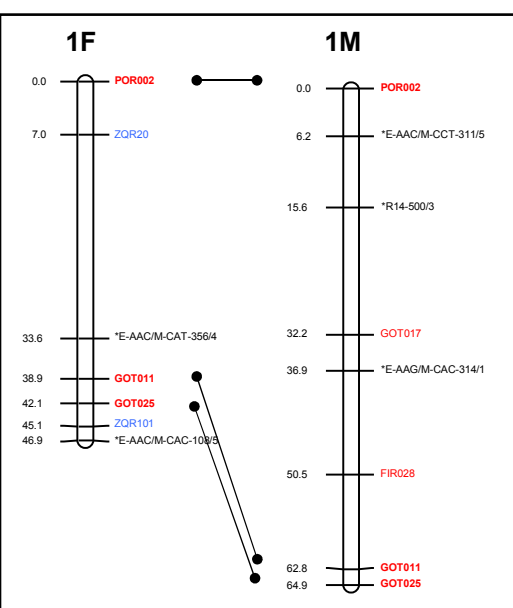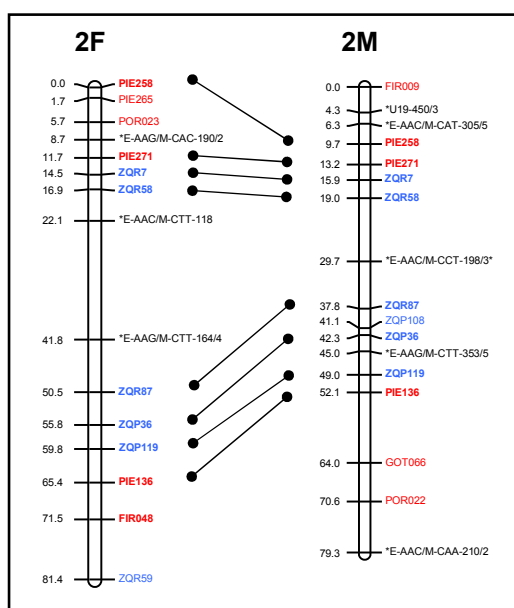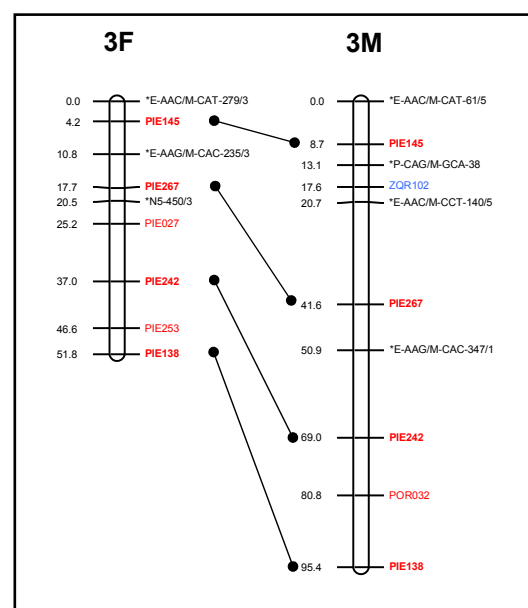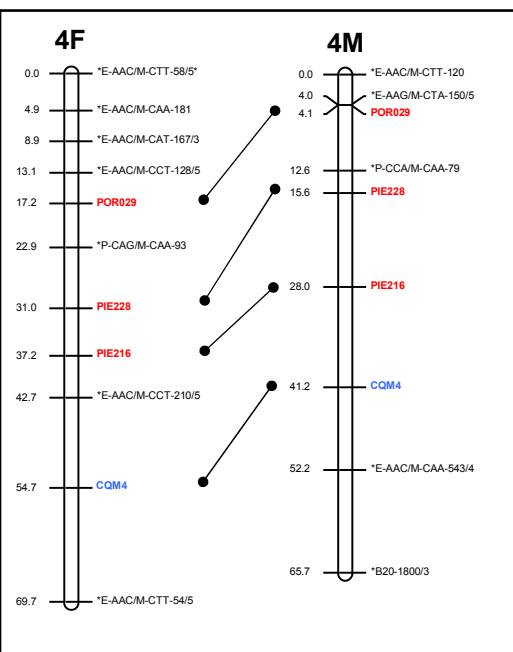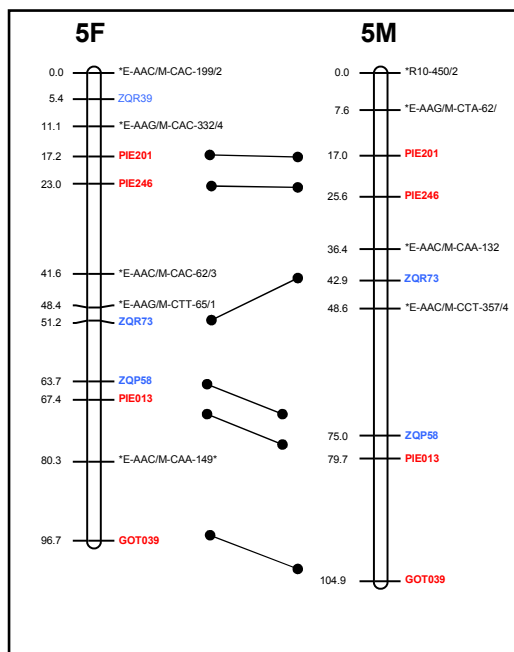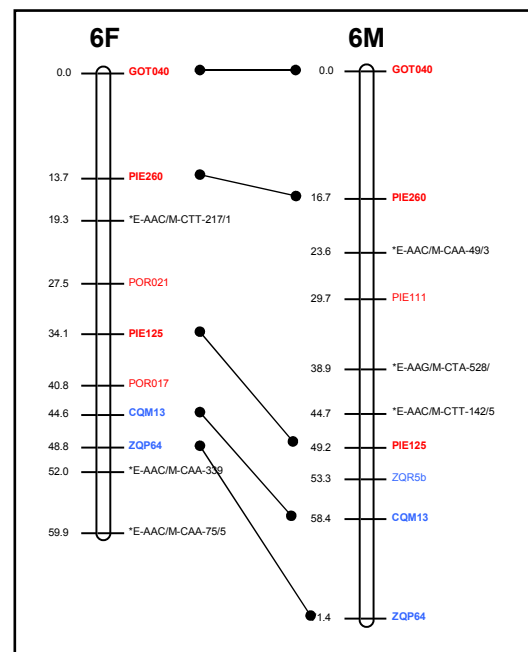

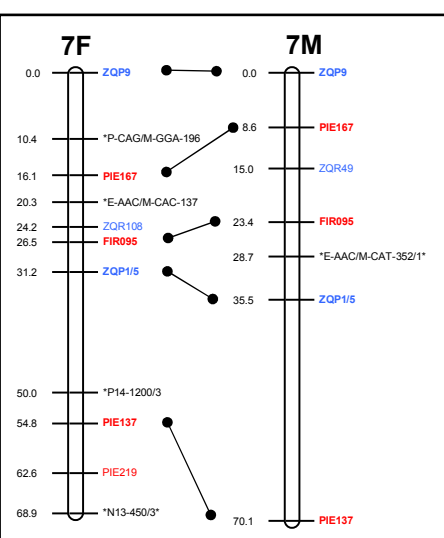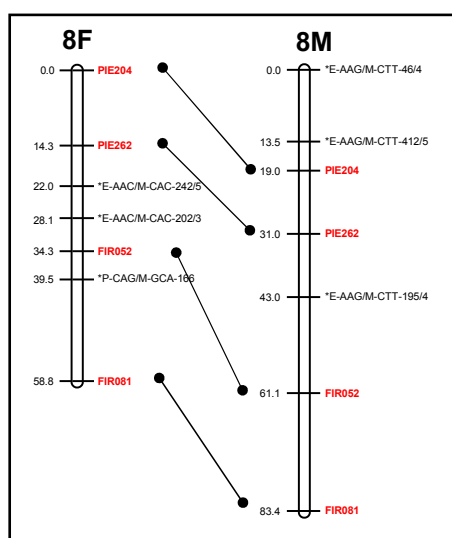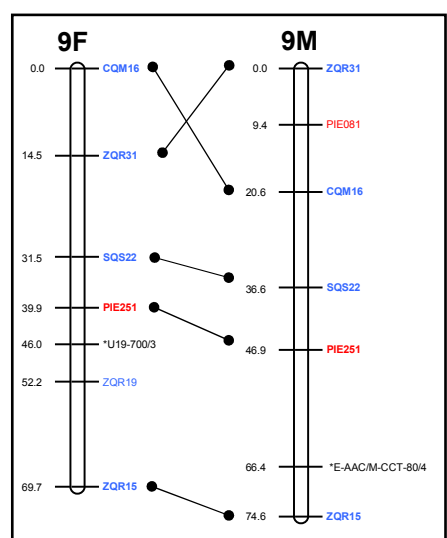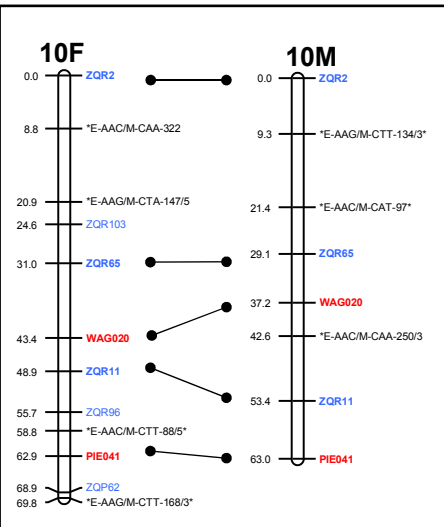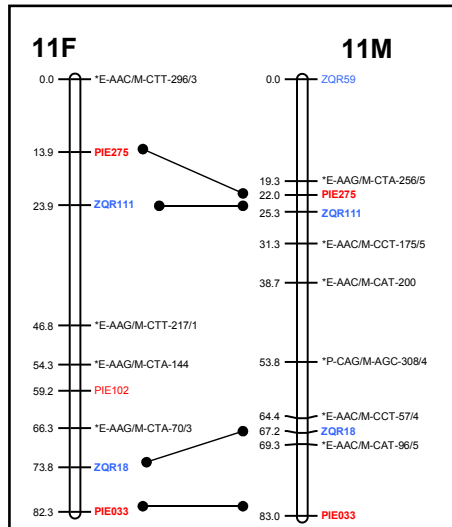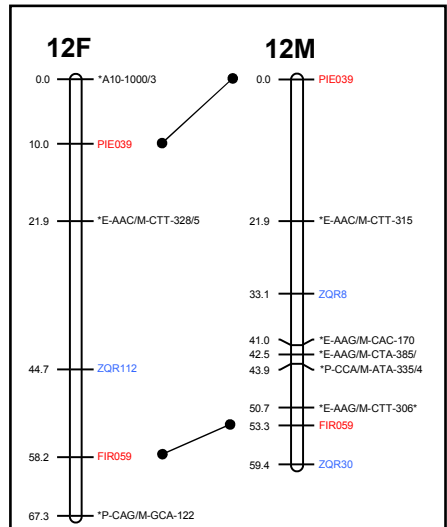

Supplement: Additional file 6 — Figure S1. A macrosynteny map for oak based on 55 intercross SSRs. In black: framework markers (AFLP, RAPD), in red: EST-SSRs, in blue: gSSRs. Bold types indicate fully informative SSRs. Female linkage groups on the left (F), male linkage group on the right (M). [file 1471-2164-11-570-S6.PDF]

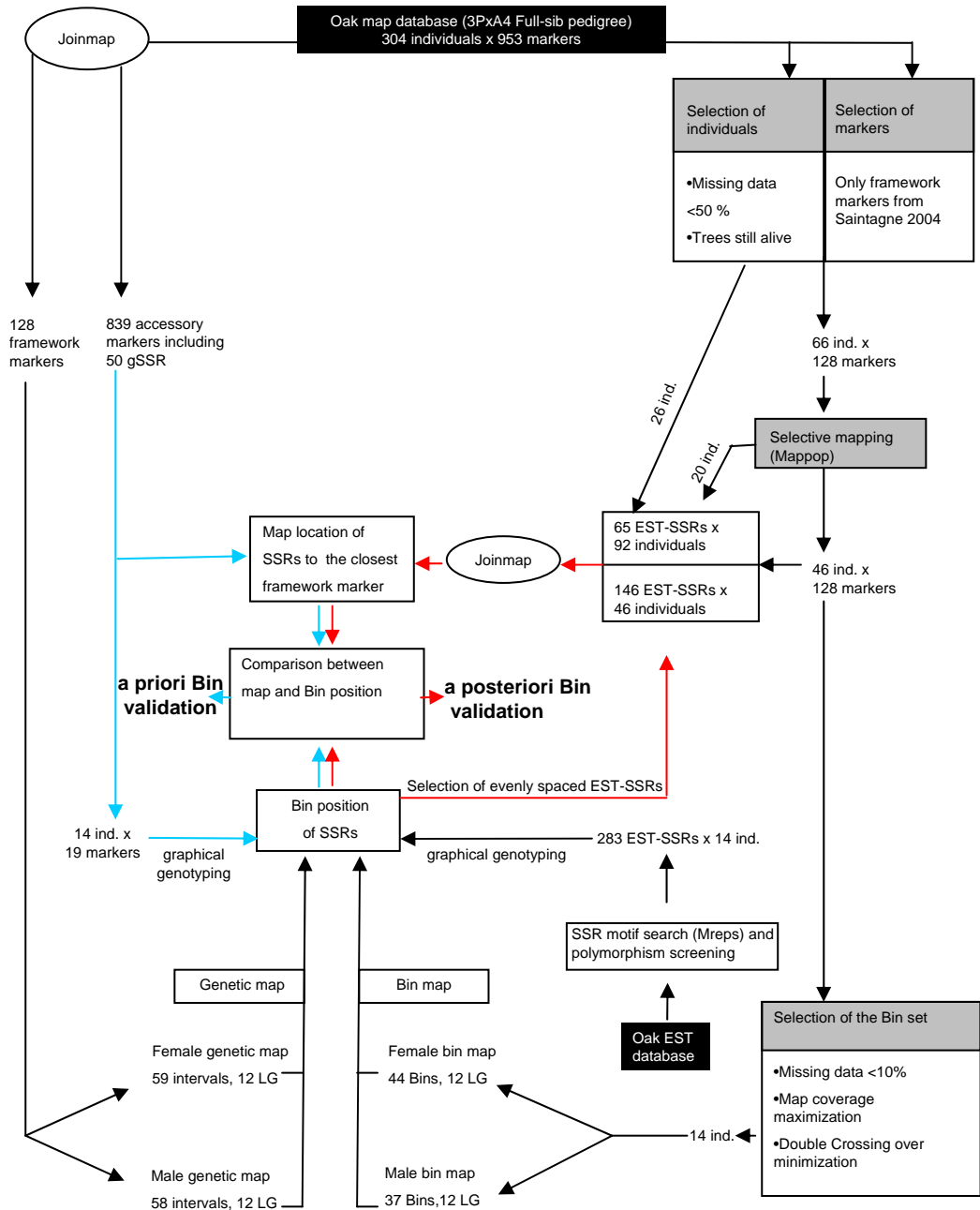

Supplement: Additional file 8 — Figure S1. Schematic representation of the bin mapping strategy. [file 1471-2164-11-570-S8.PDF]
