# Supplementary material for: A fast and cost-effective approach to develop and map EST-SSR markers: oak as a case study
Source: BMC Genomics. 2010 Oct 15;11:570. doi: 10.1186/1471-2164-11-570 (PMC3091719; doi:10.1186/1471-2164-11-570)

Figure S1a SSR location based on FrameDP

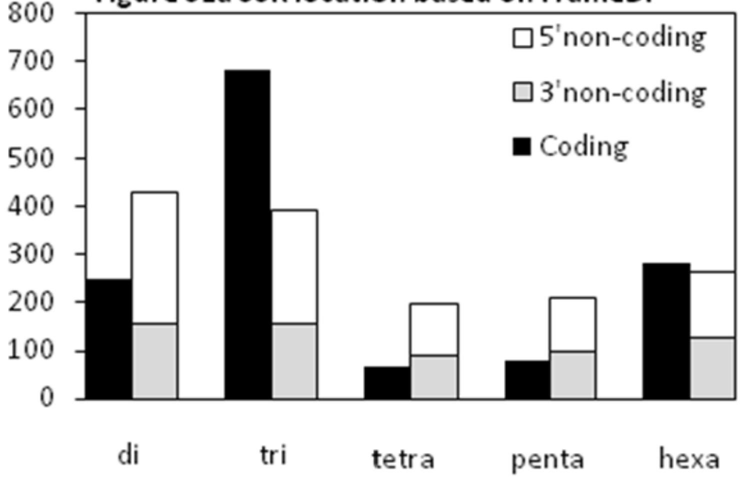

Figure S1b SSR location based on ESTScan

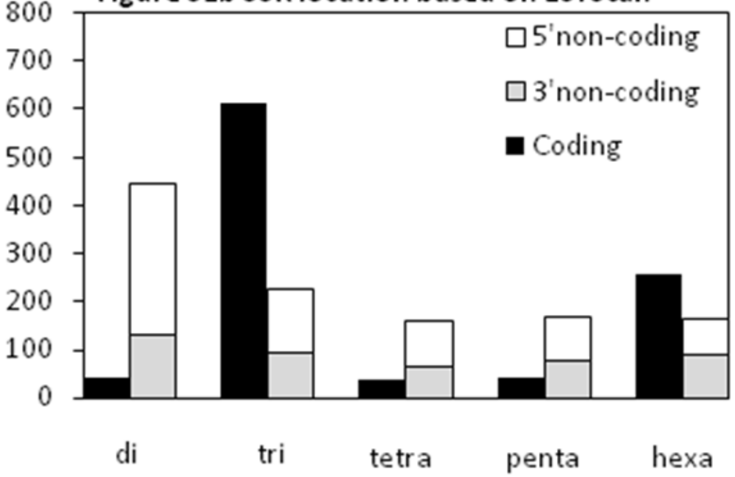

Figure S1c SSR location based on annotation

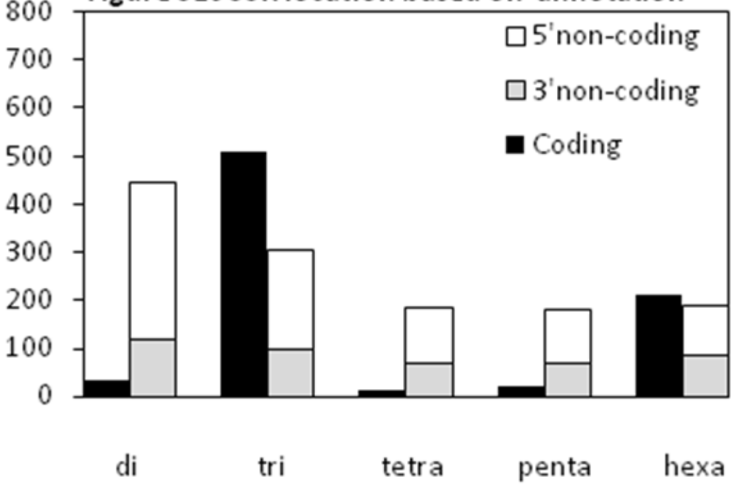

Supplement: Additional file 7 — Figure S1 Location of EST-SSRs based on FrameDP (a), ESTscan (b) and structural annotation (c) for a set of 4,664 poplar genes. Methods. 1. 4,664 full-length cDNA sequences of poplar, downloaded from Genbank. 2. SSRs searched using mreps program with default parameters. 3. Coding sequences estimated by FrameDP and ESTScan. A matrix based on Arabidopsis CDS was used for ESTScan. 4. SSR location (coding or non-coding) inferred by combining FrameDP and mreps results (Figure S1a) and ESTScan and mreps results (Figure S1b). SSR locations were also determined using mreps results and structural annotation for the corresponding cDNA (Figure S1c). Results. Figure S1a: SSR location based on the estimation by FrameDP. Figure S1b: SSR location based on the estimation by ESTScan. Figure S1c: SSR location based on structural annotation. [file 1471-2164-11-570-S7.PDF]
